# Microbial Community and Functional Gene Changes in Arctic Tundra Soils in a Microcosm Warming Experiment

**DOI:** 10.3389/fmicb.2017.01741

**Published:** 2017-09-19

**Authors:** Ziming Yang, Sihang Yang, Joy D. Van Nostrand, Jizhong Zhou, Wei Fang, Qi Qi, Yurong Liu, Stan D. Wullschleger, Liyuan Liang, David E. Graham, Yunfeng Yang, Baohua Gu

**Affiliations:** ^1^Environmental Sciences Division, Oak Ridge National Laboratory Oak Ridge, TN, United States; ^2^Department of Chemistry, Oakland University Rochester, MI, United States; ^3^State Key Joint Laboratory of Environment Simulation and Pollution Control, School of Environment, Tsinghua University Beijing, China; ^4^Department of Microbiology and Plant Biology, Institute for Environmental Genomics, University of Oklahoma Norman, OK, United States; ^5^Earth and Environmental Sciences, Lawrence Berkeley National Laboratory Berkeley, CA, United States; ^6^Research Center for Eco-Environmental Sciences, Chinese Academy of Sciences Beijing, China; ^7^Oak Ridge National Laboratory, Climate Change Science Institute Oak Ridge, TN, United States; ^8^Environmental Molecular Sciences Laboratory, Pacific Northwest National Laboratory Richland, WA, United States; ^9^Biosciences Division, Oak Ridge National Laboratory Oak Ridge, TN, United States

**Keywords:** soil organic carbon, climate warming, microbial community, functional genes, permafrost

## Abstract

Microbial decomposition of soil organic carbon (SOC) in thawing Arctic permafrost is important in determining greenhouse gas feedbacks of tundra ecosystems to climate. However, the changes in microbial community structure during SOC decomposition are poorly known. Here we examine these changes using frozen soils from Barrow, Alaska, USA, in anoxic microcosm incubation at −2 and 8°C for 122 days. The functional gene array GeoChip was used to determine microbial community structure and the functional genes associated with SOC degradation, methanogenesis, and Fe(III) reduction. Results show that soil incubation after 122 days at 8°C significantly decreased functional gene abundance (*P* < 0.05) associated with SOC degradation, fermentation, methanogenesis, and iron cycling, particularly in organic-rich soil. These observations correspond well with decreases in labile SOC content (e.g., reducing sugar and ethanol), methane and CO_2_ production, and Fe(III) reduction. In contrast, the community functional structure was largely unchanged in the −2°C incubation. Soil type (i.e., organic vs. mineral) and the availability of labile SOC were among the most significant factors impacting microbial community structure. These results demonstrate the important roles of microbial community in SOC degradation and support previous findings that SOC in organic-rich Arctic tundra is highly vulnerable to microbial degradation under warming.

## Introduction

The northern permafrost region contains over 1,600 Pg of soil organic carbon (SOC), accounting for ~50% of the estimated global below-ground organic carbon pool (Tarnocai et al., 2009). About 88% of the carbon is stored in perennially frozen soils and deposits. The SOC content varies with landscape type, from lowest in rubble land (3.4 kg SOC/m^2^) and mountain soils (3.8 kg SOC/m^2^) to highest in lowland (55.1 kg SOC/m^2^) and hilly upland soils (40.6 kg SOC/m^2^) (Ping et al., 2008). As global temperature rises, SOC stored in Arctic tundra could be readily decomposed by increased microbial activity, thereby increasing carbon dioxide (CO_2_) and methane (CH_4_) emission and resulting in a substantial positive feedback to climate change (Heimann and Reichstein, 2008). It has been predicted that global mean temperature over land could increase by 4°C by the end of the Twenty-first century (Diffenbaugh and Field, 2013), especially in polar regions, where temperature increases faster than the rest of the world (Fyfe et al., 2013).

Many studies have documented the responses of microbial communities to warming in both laboratory microcosms and field settings of tundra ecosystems (Mackelprang et al., 2011; Coolen and Orsi, 2015; Hultman et al., 2015; Bracho et al., 2016; Xue et al., 2016), but gaps remain with regard to how warming would affect microbial community functional structure in relation to SOC degradation, methanogenesis, and iron reduction in anoxic tundra soils. Temperature is generally considered a key limiting factor for microbial metabolism. Because microbial communities and their ability to degrade SOC have different temperature sensitivities, even small changes in microbial diversity or functional gene abundances could have significant influences on SOC stock and degradation (Davidson and Janssens, 2006; Cardenas et al., 2015). Additionally, water saturation is linked to the quantities and relative proportions of CO_2_ and CH_4_ released from these soils (Lipson et al., 2012). At lowland locations, such as trough areas of tundra polygons, soils are usually water saturated, which limits oxygen diffusion but permits anaerobic metabolisms such as fermentation, iron reduction, and methanogenesis (Sturtevant and Oechel, 2013; Herndon et al., 2015; Yang et al., 2016). In dryer uplands, soil oxygen drives aerobic respiration, leading to SOC decomposition, primarily to CO_2_ (Zona et al., 2011).

Anaerobic iron metabolism also contributes significantly to iron redox cycling in water and sediments (Weber et al., 2006). Moreover, interactions between SOC and Fe(III)-oxyhydroxide minerals strongly influence SOC degradation, soil respiration, and CH_4_ production in anoxic, water-saturated soils. Fe(III) or Fe(III)-oxyhydroxides not only serve as terminal electron acceptors during anaerobic microbial respiration but also form complexes with soil organic C, thereby decreasing SOC bioavailability for degradation (Gu et al., 1994; Baldock and Skjemstad, 2000; Kleber et al., 2005; Lalonde et al., 2012). However, Fe(III) reduction and SOC desorption from mineral surfaces could make SOC more bioavailable for degradation. Anaerobic respiration in shallow peat soils on the Arctic coastal plain is dominated by Fe(III) reduction (Lipson et al., 2012). Conversely, iron-oxidizing bacteria can convert reduced Fe(II) to oxidized Fe(III) along with SOC decomposition downstream (Hall and Silver, 2013; Emerson et al., 2015). It is thus critical to understand the interconnections between microbial communities involved in SOC degradation, methanogenesis, and iron reduction, and to uncover the environmental variables affecting these interactions under warming conditions.

Temperature increase can stimulate microbial SOC degradation, but certain groups of bacteria may be stimulated more than others and thereby induce changes in microbial community functional structure. As SOC resources deplete over time, functional genes associated with SOC decomposition also decrease. We therefore postulate that genes associated with C cycling and Fe(III) reduction will decline as SOC bioavailability decreases. Accordingly, the present study was designed to: (1) assess warming effects on the abundance of microbial functional genes and the structure of microbial communities in an Arctic soil; (2) identify the relationships between environmental variables (e.g., soil type, temperature, incubation time) and the functional genes associated with SOC degradation; and (3) examine how warming affects microbial genes, especially those related to CH_4_ production and Fe(III) reduction. The functional gene array GeoChip 5.0 was utilized as a platform in which important bacterial, archaeal, and fungal functional genes (with >16,000 probes) can be detected, even at low abundance (with ≤10 pg of DNA template) (He et al., 2007; Zhou et al., 2012). The probes are specific and quantitative for determining the relative abundance of microbial functional genes in different soils from diverse environmental settings (He et al., 2007, 2010, 2012).

## Materials and methods

### Soil samples and incubation setup

Soil samples and incubation setups were the same as those used in a previous study of warming effects on organic carbon degradation in an Arctic tundra soil (Yang et al., 2016). Briefly, soil cores were taken in April 2012 from the trough area of a high-centered polygon in a continuous permafrost and interstitial tundra region (N 71°16.757′ W 156 °36.274) at the Barrow Environmental Observatory (BEO), Barrow, Alaska, USA (Yang et al., 2016). The average annual temperature at the BEO is −12.6°C, and annual precipitation is 114 mm (Hubbard et al., 2013). Soils in the BEO are generally classified as Gelisols, characterized by an organic-rich surface layer underlain by a horizon of silty clay or silt loam-textured mineral material, and a frozen organic-rich mineral layer. Soil cores were kept frozen in sealed PVC liners during shipping, and stored at −20°C until the day of processing. Core processing and microcosm incubation design are described in detail elsewhere (Herndon et al., 2015; Roy Chowdhury et al., 2015; Yang et al., 2016). In brief, organic-rich (8–20 cm below the land surface) and mineral-rich (22–45 cm) soils were homogenized separately in a N_2_-filled anoxic glove chamber. Anoxic microcosms were constructed to mimic the water-logged active layer of permafrost-affected soils, as the average thaw depth at this location is about 45 cm. The homogenized wet soils (150 g) were placed into 600-mL sterile bottles inside the glove chamber. A relatively high headspace/soil volume (>5:1) was used in the incubation, allowing sufficient exchange of soil gases to minimize the bottle effect. Two incubation temperatures (−2°C and 8°C) were chosen to simulate a near-freezing condition and the warmest month at the BEO (Roy Chowdhury et al., 2015). Triplicate samples (*n* = 3) per soil type per incubation temperature were conducted. Selected soil subsamples were taken at 0, 34, and 122 days (at −2°C) and at 0, 60, and 122 days (at 8°C) based on instrument availability. The headspace was flushed with ultra-pure N_2_ to replace the produced CO_2_ and CH_4_ gases after each sampling event, mimicking natural field conditions. The samples were kept at −20°C prior to GeoChip and geochemical analyses.

### Geochemical analyses

Extractable SOC, inorganic chemical species, CO_2_ and CH_4_ were analyzed, as described previously (Yang et al., 2016). In short, headspace CO_2_ and CH_4_ were analyzed with a SRI 8610C gas chromatograph equipped with a flame ionization detector (SRI Instruments, Torrance, CA). Soil samples were extracted with either 0.1 M KCl (pH ~ 5.0) or 10 mM NH_4_HCO_3_ (pH ~ 7.3) solution to determine soluble soil organic compounds and dissolved inorganic species. Total dissolved organic C (DOC) was measured by a Shimadzu TOC-L analyzer (Shimadzu Corp., Kyoto, Japan). Alcohol compounds were analyzed with an Agilent gas chromatograph (Agilent Technologies), and organic acids were measured by ion chromatography using a Dionex DX500 system (ThermoFisher Scientific, Madison, WI). Simple sugars such as glucose and cellobiose were analyzed on a high-performance liquid chromatograph (Waters, Milford, MA). Dissolved Fe(II) and total Fe concentrations were quantified using phenanthroline method following the HACH procedures 8146 and 8008, respectively, on a HACH DR 900 colorimeter.

### GeoChip hybridization and data analyses

GeoChip 5.0 (with >16,000 probes) was used for quantitative analyses of bacterial, archaeal, and fungal functional genes. It has been shown to provide a linear relationship (*r* = 0.89–0.99) between target DNA and hybridization signal intensities over 8 ng to 1 μg DNA from pure cultures, mixed cultures, and environmental samples with or without amplification (He et al., 2007, 2010; Zhou et al., 2012). Soil DNA was extracted by freeze-grinding mechanical lysis, and purified using a low melting agarose gel followed by a phenol extraction (Zhou et al., 1996). The purified DNA (1 μg) was labeled with Cy3 dye (GE Healthcare Life Sciences, Pittsburg, PA), dried, rehydrated and hybridized with GeoChip 5.0, as described previously (Zhou et al., 2012; Wang et al., 2014). GeoChip microarrays were then washed and scanned with a NimbleGen MS 200 Microarray Scanner (Roche, Switzerland) at 100% photomultiplier and 100% laser power. Array spots with a signal-to-noise ratio of less than 2.0 were deleted before data processing.

Signal intensities were standardized, and GeoChip data were processed using the following steps (Liu et al., 2015): (i) remove genes detected in only one of three replicate samples; (ii) transform signal intensity data into the logarithmic format; and (iii) normalize the intensity of each probe by dividing the probe intensity by the mean intensity of the microarray. Data were presented as the total relative gene abundance (Dimensionless), calculated by summarizing the relative abundances of all functional genes detected in each gene category.

Detrended correspondence analysis (DCA) was used to determine changes in the overall microbial functional structure. Bray-Curtis and Jaccard distances were used to calculate dissimilarity matrices from GeoChip data (Yue et al., 2015). Dissimilarity tests, including Adonis, Anosim, and MRPP, were performed to calculate the differences among different soil and temperature treatments. Mantel tests and canonical correspondence analysis (CCA) were conducted to correlate environmental variables with microbial functional structure. All analyses were conducted in R (v.3.1.1) software with functions from the Vegan package (v.1.15-1) (He et al., 2007).

## Results and discussion

### Overall functional structural differences between the organic and mineral soils

**Figure 1:**
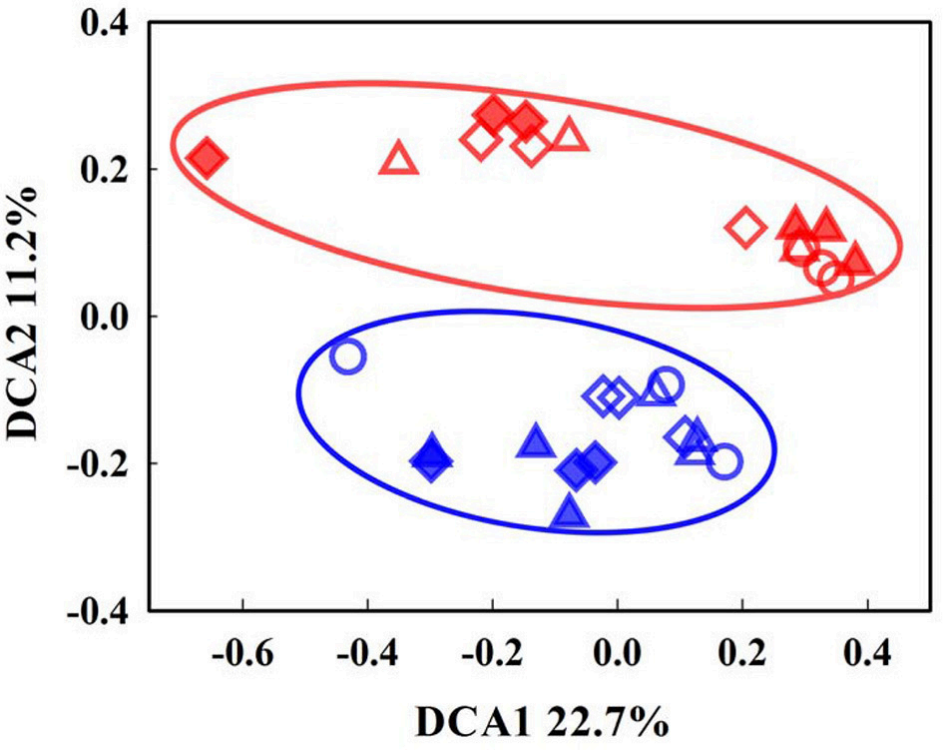
Detrended correspondence analysis (DCA) of microbial community patterns between the organic- and mineral-rich soils. The data include both pre-incubation and incubated samples at −2 and 8°C. Open circles, triangles, and diamonds represent samples incubated at −2°C for 0, 34, and 122 days, respectively. Solid triangles and diamonds represent samples incubated at 8°C for 60 and 122 days. Organic-rich samples are marked red and mineral soils marked blue.

**Figure 2:**
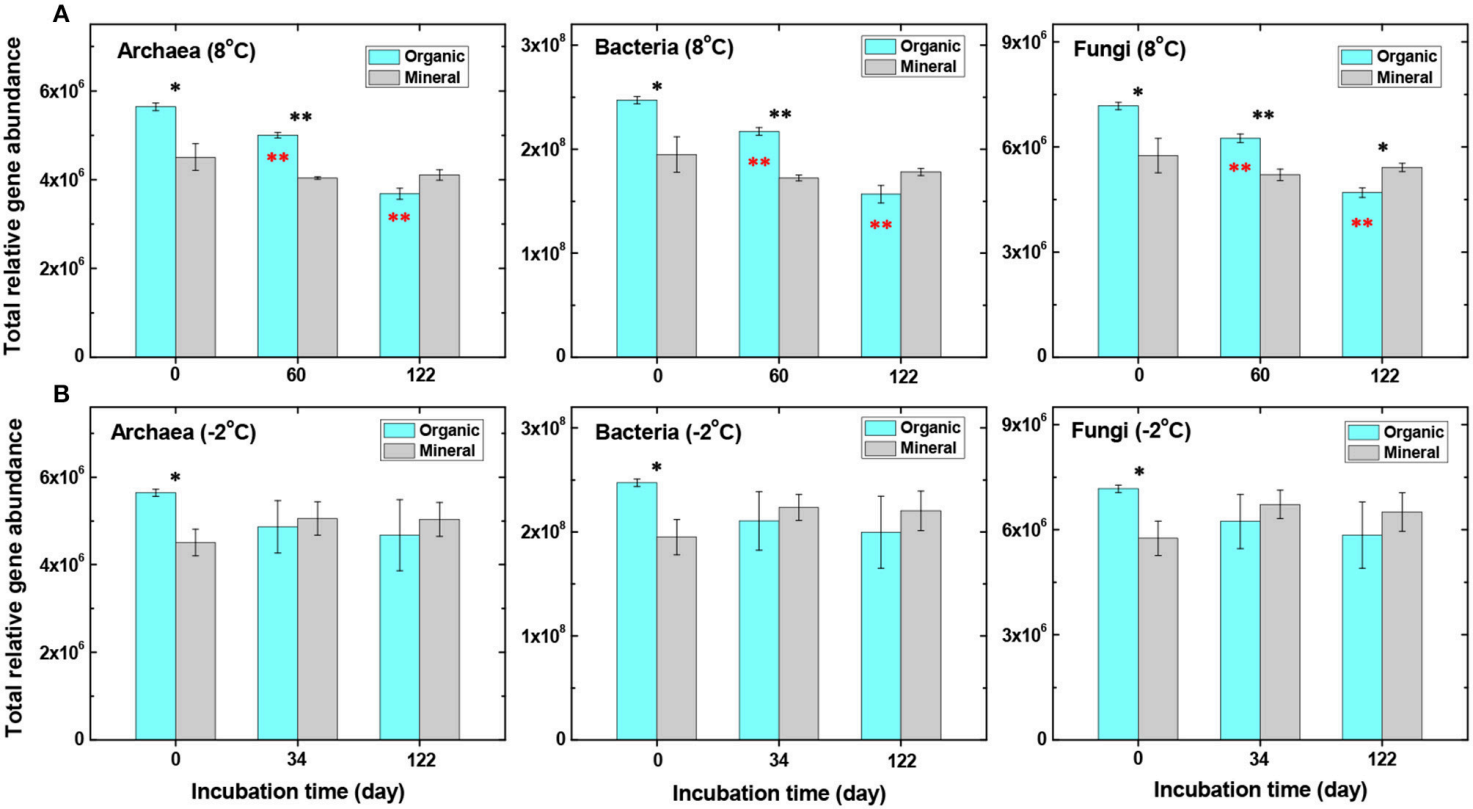
Comparisons of total relative gene abundance of archaeal, bacterial, and fungal genes in the organic and mineral soils. Soil samples were incubated at either 8°C **(A)** or −2°C **(B)** from day 0 to day 122 (data analyzed by *t*-test, ^*^P < 0.05 and ^**^P < 0.01). Black stars represent significant differences between organic and mineral soils, and red stars represent significant differences between organic soil samples before (time 0) and after incubation (at 60 and 122 days).

The overall functional structure of the microbial communities was examined in soils before and after incubation at either −2 or 8°C. Distinct patterns of the communities were observed between the organic and mineral soils in the DCA ordination plot of the GeoChip data (Figure 1), suggesting that the communities were well separated based on soil type. Prior to incubation (Day 0), total amounts of archaeal, bacterial, and fungal genes in the mineral soil were lower (*P* < 0.05) than those in the organic soil (Figure 2). Dissimilarity tests, including Adonis, Anosim, and MRPP, further verified significant differences (*P* < 0.05) in microbial communities between the two soil types (Table S1).

Higher abundances of microbial communities observed in the organic-rich soil were consistent with higher production rates of CO_2_ and CH_4_ in the organic than the mineral soils during incubation at 8°C (Yang et al., 2016), since the organic soil contained ~22.5 ± 1 mmol C g^−1^ dwt soil, nearly twice as high as the mineral soil (11.6 ± 1 mmol C g^−1^ dwt). In particular, labile organic C (e.g., DOC, extractable acetate and alcohol) concentrations were much higher in the organic soil than in the mineral soil (Table S2). The extractable acetate concentration (18.1 ± 1.8 μmol g^−1^ dwt) was roughly 30 times higher in the organic than in the mineral soil (Table S2). The organic soil also contained a higher gravimetric water content (~350%) than the mineral soil (~90%) supporting anaerobic respiration and CH_4_ production (Lipson et al., 2012). These observations agreed well with that reported in a previous study showing that microbial abundance was much higher in organic than mineral layers of Arctic soils (Yergeau et al., 2010).

Total relative abundances of archaeal, bacterial, and fungal genes generally decreased with incubation time (from 0 to 60 and to 122 days) at 8°C, but less so in the mineral soil (Figure 2A). The decrease was the most significant (*P* << 0.01) in the organic soil after 122 days of incubation, when gene abundance decreased to a level even lower than that in the mineral soil (Figure 2A). At −2°C, the total abundances decreased in the organic soil, but increased slightly in the mineral soil (Figure 2B), although they are not statistically significant (*P* > 0.05). Functional diversity of microbial communities, calculated by the Shannon index (Leff et al., 2015), was also lower at 8°C than at −2°C in the organic soil (*P* < 0.05) (Table 1). However, no significant changes in the Shannon index were observed in the mineral soil under the same experimental conditions. Warming at 8°C thus decreased the microbial functional diversity in the organic soil, but not in the mineral soil.

**Table 1:** Shannon index showing functional diversity in the organic and mineral soils incubated at either −2 or 8°C.

**Soil layer**	**Day 0**	**Incubated at** −**2°C**		**Incubated at 8°C**	
		**Day 34**	**Day 122**	**Day 60**	**Day 122**
Organic soil	11.10	10.86	10.87	11.10	10.62^*^
Mineral soil	10.88	11.02	10.96	10.82	10.84
*P*-value (Organic vs. mineral)	0.22	0.29	0.37	**0.01**	0.30

* *P < 0.05 by t-test, and values marked bold*.

A decrease in microbial community functional diversity at 8°C is not surprising because microbial respiration and growth rates are temperature dependent (Luo et al., 2001; Rinnan et al., 2007; Bradford et al., 2008; Frey et al., 2008). An increase in temperature is expected to stimulate microbial activity and thus rapid turnover of SOC (Mikan et al., 2002; Lavoie et al., 2011; Graham et al., 2012). Additionally, soil water in thawed permafrost not only accelerates the diffusion of substrates, nutrients, and microbial products (Romanovsky and Osterkamp, 2000), but also increases microbial growth and activity (Mikan et al., 2002).

As stated earlier, the organic soil contains much higher amounts of labile SOC substrates (e.g., acetate and ethanol) than the mineral soil (Table S2) (Yang et al., 2016) to support microbial growth. In the mineral soil, however, SOC could be conserved by organo-mineral associations (Kleber et al., 2007). Thus, the availability of abundant labile organic substrates in the organic soil may stimulate fast-growing microbial taxa to flourish and change the overall community composition. This would explain higher total relative abundances of functional genes observed in the organic soil than in the mineral soil at 8°C. However, in the late stage of incubation (122 days), despite high availability of labile SOC, the microbial diversity may be suppressed in the organic layer due to limited availability of other essential nutrients such as N and P, as previously observed (Luo et al., 2004; Reich et al., 2006; Pautler et al., 2010; Sistla and Schimel, 2013). Similar findings were reported in Antarctic soils, and the effect was attributed to a shift toward generalist bacterial communities upon warming (Yergeau et al., 2012). Similarly, a microbial community shift, characterized by increase in Gram positive bacterial biomass, was reported in a low Arctic tundra after soils were thawed (Buckeridge et al., 2013; Koyama et al., 2014; Xue et al., 2016).

Changes in the total abundances of archaeal, bacterial and fungal genes at −2°C were not statistically significant (*P* > 0.05) in both organic and mineral soils (Figure 2B and Table 1), possibly as a result of lower microbial activity at −2°C. However, gene abundance in the mineral soil appeared slightly higher than in the organic soil after 34 and 122 days of incubation (Figure 2B). This result could explain a slightly higher production rate of CH_4_ or a higher CH_4_/CO_2_ rate ratio, previously observed in the mineral soil than the organic soil at −2°C (Yang et al., 2016). A possible explanation is that, due to overall lower organic C substrate availability in the mineral soil, the release of even small amounts of organic C or nutrients previously immobilized within mineral matrixes upon warming at −2°C (relative to −20°C) could potentially result in a greater stimulation of the microbial community in the mineral soil than in the organic soil.

### Linkages between environmental variables and functional community structure

To identify whether there was a relationship between environmental factors and microbial functional genes, a Mantel test was carried out with 10 selected environmental variables, including soil type (organic vs. mineral), incubation time, temperature, DOC, sugars, ethanol, acetate, CH_4_, CO_2_, and Fe(II)/Fe(total) ratio, as input parameters. Soil pH was nearly constant, being 7.2 ± 0.1 in the organic soil and 7.3 ± 0.1 in the mineral soil. As shown in Table 2 and Table S3, soil type, DOC, and acetate concentrations were found to have the most significant impact on the functional genes as a whole, with *P* < 0.04, normalized by the Benjamini-Hochberg method. The DOC concentration in particular showed the most significant impact by CCA analysis (*P* = 0.001, Figure S1). These results were in general agreement with the observation that labile SOC (e.g., reducing sugars) in the water-extractable carbon pool was quickly consumed in the early stage of the incubation (Yang et al., 2016), whereas fermentation products such as ethanol and acetate were subsequently converted to CH_4_ and CO_2_. We also found that soil DOC and acetate were significantly correlated with genes that are associated with C metabolism (i.e., C-cycling genes in the text below) (*P* < 0.05) (Table 2), suggesting that microbial functional genes, particularly C cycling genes in the Arctic soil, were closely associated with the distribution of labile SOC pools. This finding is in line with recently observed relationships between the expressed gene-enzyme and C dynamics, indicating important roles of microbes in regulating soil carbon cycles (Zhao et al., 2014; Liu et al., 2015; Trivedi et al., 2016).

**Table 2:** Relationships between ten environmental variables (i.e., soil layer, incubation time, temperature, DOC, reducing sugar, ethanol, acetate, CH_4_, CO_2_, and Fe(II)/Fe(total)) and total functional genes and C-cycling genes.

**Genes**	**Total functional genes**		**C-cycling genes**	
**Simple mantel**	***r***	***P***	***r***	P^*^
Soil layer	0.147	**0.025**	0.155	**0.025**
Time	0.094	0.088	0.094	0.078
Temperature	0.069	0.104	0.071	0.101
DOC	0.158	**0.025**	0.166	**0.025**
Reducing sugar	0.088	0.172	0.093	0.151
Ethanol	0.052	0.250	0.053	0.270
Acetate	0.273	**0.040**	0.277	**0.033**
CH_4_	0.175	0.104	0.175	0.090
CO_2_	0.248	0.088	0.247	0.078
Fe(II)/Fe(total)	0.121	0.105	0.122	0.111

* *P-values were adjusted by the Benjamini-Hochberg method using sequentially modified Bonferroni correction for multiple hypothesis testing and marked bold at P < 0.05*.

**Figure 3:**
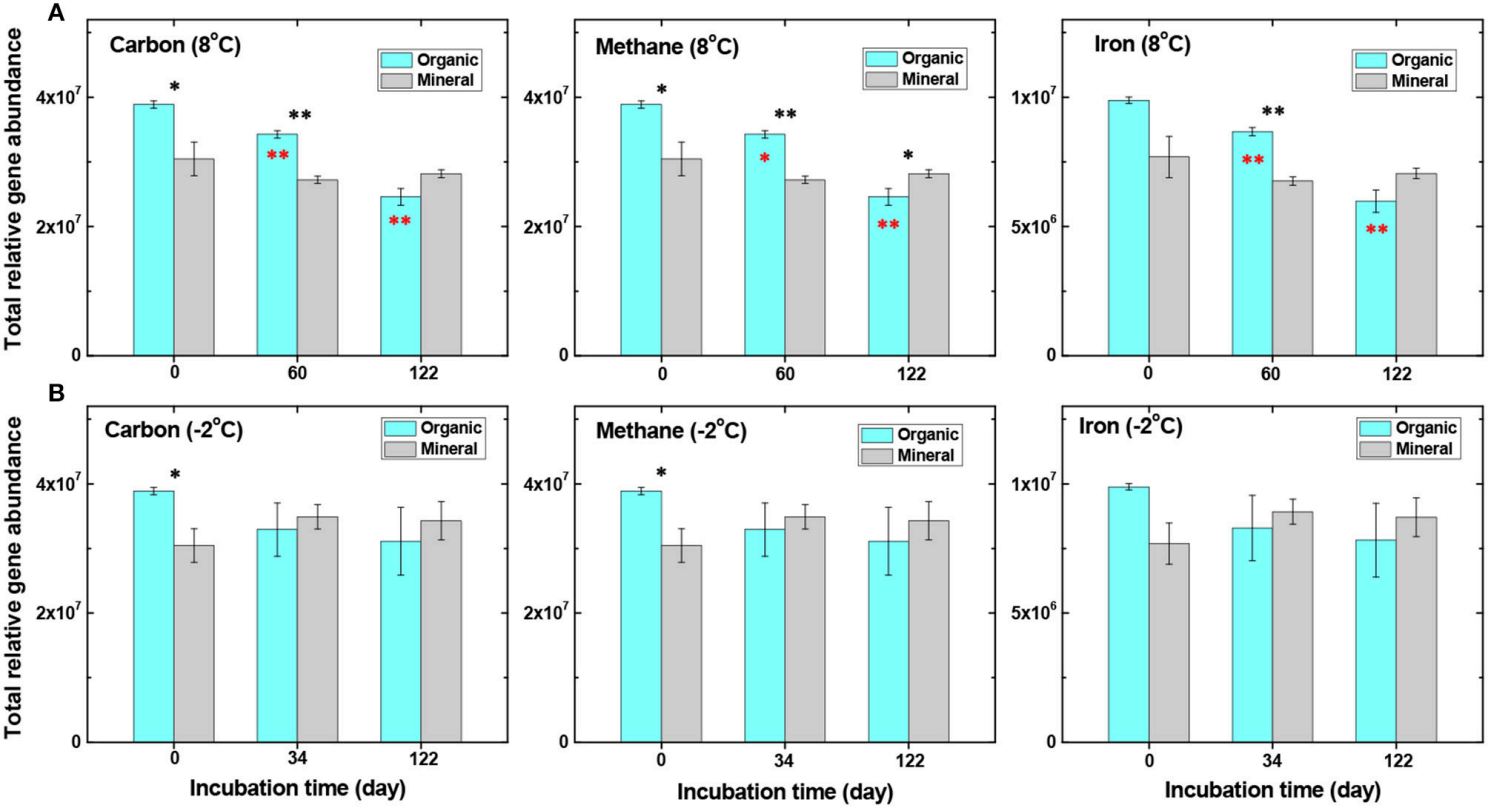
Comparisons of total relative gene abundance related to carbon cycling, CH_4_ production/degradation, and iron cycling. The organic and mineral soils were incubated at either 8°C **(A)** or −2°C **(B)** from day 0 to day 122 (data analyzed by *t*-test, ^*^P < 0.05 and ^**^P < 0.01). Black stars represent significant differences between organic and mineral soils, and red stars represent significant differences in the organic soil (in comparison to pre-incubation samples).

We further examined the abundance of C-cycling genes related to the degradation of SOC. Incubations at a near-freezing temperature (−2°C) resulted in little change in the total abundance of C-cycling genes in both organic and mineral soils (Figure 3 and Table 3). This is expected because near-freezing temperatures may uncouple the linkages between organic substrate quality or C chemistry and the temperature dependence of microbial respiration (Mikan et al., 2002). At 8°C, however, the total abundance of C-cycling genes in the organic soil decreased progressively from Day 0 to Day 60 and continued to Day 122 (*P* < 0.05) (Figure 3A), whereas gene abundance in the mineral soil was not significantly changed. Genes that encode enzymes for lignin and aromatic compounds were substantially less abundant in organic soil after 8°C incubation, including *manganese peroxidase, limonene epoxide hydrolase, glyoxal oxidase*, and *vanillin dehydrogenase* (Table 3), likely in response to incubation under anoxic conditions. These degradation processes require oxygen and may have played key roles in C degradation by regulating oxygen-independent C hydrolases (Freeman et al., 2001).

**Table 3:** Total relative gene abundance related to carbon degradation, fermentation, CH_4_ cycling, and iron reduction and uptake in the organic soil after incubation at either −2°C or 8°C.

**Genes (in thousands)**	**Preincubation soil**	**Incubated at −2°C**		**Incubated at 8°C**	
		**Day 34**	**Day 122**	**Day 60**	**Day 122**
**CARBON DEGRADATION (LABILE TO RECALCITRANT FROM STARCH TO LIGNIN)**					
**Starch**					
amyA	8, 245.2 ± 98.0	6, 910.7 ± 943.0	6, 504.1 ± 1102.2	7, 350.2 ± 112.4^**^	5, 183.3 ± 375.5^**^
glucoamylase	423.4 ± 2.7	367.9 ± 65.5	331.8 ± 47.2	380.2 ± 8.0^**^	260.0 ± 19.3^**^
pula	368.1 ± 2.7	307.9 ± 42.1	281.8 ± 60.4	329.9 ± 5.7^**^	226.3 ± 9.6^**^
**Hemicellulose**					
ara	1, 187.6 ± 16.2	1, 070.0 ± 87.3	1, 020.9 ± 152.9	1, 032.0 ± 14.2^**^	839.8 ± 14.9^**^
xyla	634.8 ± 21.4	524.5 ± 86.5	474.8 ± 105.6	**571.7 ± 15.2**	372.3 ± 22.8^**^
xylanase	910.9 ± 17.6	751.1 ± 112.6	700.4 ± 114.8	812.2 ± 12.9^*^	546.4 ± 55.8^**^
**Cellulose**					
cellobiase	738.0 ± 6.6	580.3 ± 101.3	534.6 ± 101.4	650.0 ± 8.5^**^	399.3 ± 35.1^**^
endoglucanase	600.0 ± 4.8	500.9 ± 85.0	459.4 ± 86.5	559.8 ± 10.2^*^	354.4 ± 25.8^**^
exoglucanase	178.2 ± 4.9	145.4 ± 27.5	136.3 ± 27.1	144.5 ± 3.6^**^	92.4 ± 11.3^**^
**Chitin**					
Acetylgluco- saminidase	1, 374.9 ± 19.0	1, 153.8 ± 161.1	1, 079.8 ± 209.4	1, 236.2 ± 18.1^**^	826.2 ± 58.3^**^
chitinase	2, 557.5 ± 46.8	2, 166.2 ± 279.2	2, 058.5 ± 352.0	2, 358.9 ± 22.3^*^	1, 641.9 ± 98.0^**^
**Aromatic**					
limeh	254.4 ± 3.7	196.2 ± 39.7	178.9 ± 41.0	211.5 ± 5.7^**^	128.4 ± 13.0^**^
vdh	94.5 ± 1.7	76.7 ± 9.8	74.3 ± 14.2	83.0 ± 2.1^*^	50.4 ± 5.2^**^
**Lignin**					
glx	120.8 ± 0.9	93.1 ± 12.5	89.2 ± 17.6	106.4 ± 0.6^**^	65.1 ± 40^**^
mnp	172.1 ± 2.4	141.6 ± 22.4	133.1 ± 22.1	144.0 ± 4.4^**^	105.4 ± 9.4^**^
phenol_ oxidase	553.5 ± 6.3	451.9 ± 69.3	423.7 ± 74.2	513.2 ± 8.0^*^	326.6 ± 34.5^**^
**Fermentation**					
AceA	564.8 ± 7.3	456.8 ± 73.9	436.6 ± 98.1	503.2 ± 13.3^*^	311.5 ± 27.2^**^
AceA_fungi	14.2 ± 0.2	11.4 ± 2.4	10.9 ± 2.7	11.4 ± 0.4^*^	6.5 ± 1.1^**^
AceB	1, 137.4 ± 19.1	926.2 ± 141.9	876.6 ± 178.2	956.6 ± 21.8^*^	621.0 ± 45.1^**^
AceB_fungi	25.1 ± 0.5	21.1 ± 3.6	19.2 ± 3.8	21.0 ± 0.4^*^	13.0 ± 2.3^**^
aclb	54.3 ± 0.7	42.9 ± 10.5	39.6 ± 9.1	52.2 ± 0.7	27.1 ± 3.4^**^
AcnA	43.9 ± 1.0	34.3 ± 5.1	34.5 ± 8.6	35.3 ± 0.9^**^	22.6 ± 0.9^**^
frdA_rTCA	67.8 ± 0.6	55.6 ± 16.0	48.5 ± 7.7	62.1 ± 4.4	35.4 ± 3.8^**^
mdh	115.8 ± 5.4	85.4 ± 11.3	95.4 ± 27.3	101.1 ± 1.7	65.0 ± 1.4^**^
sucD	18.8 ± 0.4	14.8 ± 3.3	12.3 ± 2.6	18.7 ± 0.9	10.1 ± 1.4^**^
oorA	12.5 ± 0.3	11.2 ± 1.6	11.1 ± 2.3	11.9 ± 0.8	7.3 ± 0.2^**^
pgk	436.1 ± 5.9	353.4 ± 56.1	326 ± 64.0	389.4 ± 6.7^*^	255.3 ± 21.1^**^
PRI	575.5 ± 5.6	467.7 ± 75.3	449.3 ± 73.9	504.6 ± 10.1^*^	338.8 ± 28.8^**^
TIM	822.4 ± 19.3	726.7 ± 64.1	725.2 ± 102.1	688.6 ± 13.9^*^	555.5 ± 39.3^*^
tktA	1, 366.7 ± 20.4	1, 185.9 ± 137.3	1, 139.7 ± 193.8	1, 148.0 ± 27.7^*^	873.7 ± 51.8^*^
**CH**_4_ **cycling / methanogenesis**					
cdhC methane	1.3 ± 0.1	1.1 ± 0.1	1.4 ± 0.1	1.0 ± 0.1^*^	0.7 ± 0.01^**^
fmdB/fwdB	119.6 ± 2.6	103.4 ± 11.1	97.6 ± 16.8	110.5 ± 0.8	84.3 ± 1.1^**^
ftr	82.8 ± 0.8	65.7 ± 13.1	61.0 ± 10.4	72.3 ± 1.2	47.6 ± 4.5^**^
hdrB	346.3 ± 11.8	322.6 ± 23.4	314.4 ± 47.6	289.4 ± 6.6	253.3 ± 0.6^**^
Hmd	2.0 ± 0.1	1.0 ± 0.6	1.0 ± 0.4	2.1 ± 0.1	0.8 ± 0.2^**^
Mch_methane	47.1 ± 0.4	37.5 ± 7.3	35.5 ± 7.9	41.6 ± 1.5	26.7 ± 1.9^**^
mcrA	246.0 ± 8.7	269.5 ± 17.9	275.0 ± 15.4	**215.2** ± 5.7^*^	259.2 ± 10.1
Mer_methane	27.0 ± 0.6	22.5 ± 3.7	21.1 ± 4.6	26.4 ± 0.8	16.5 ± 1.3^**^
mrtH	63.9 ± 1.3	55.2 ± 11.9	50.3 ± 5.8	52.5 ± 2.5	**35.4 ± 4.0**
MT2	4.7 ± 0.7	3.2 ± 1.0	3.1 ± 2.0	5.4 ± 0.5	2.1 ± 0.4^**^
mtaB	9.8 ± 0.2	7.3 ± 0.9	6.8 ± 0.8	9.8 ± 0.1	5.2 ± 0.3^**^
mtbC/mttC	7.2 ± 0.5	6.2 ± 1.3	5.1 ± 1.4	8.0 ± 0.3^*^	5.3 ± 0.04^**^
mtmB	5.8 ± 0.1	3.5 ± 0.1	3.4 ± 0	6.0 ± 0.01	1.5 ± 0^**^
mttB	0.2 ± 0.1	0.07 ± 0.1	0.0 ± 0.0	0.1 ± 0.06	**0 ± 0.0**
mtxX	3.4 ± 0.1	**1.9** ± 0.5^*^	2.0 ± 0.7	2.4 ± 0.06	1.0 ± 0.5^*^
**Methane oxidation**					
mmox	36.2 ± 1.3	28.7 ± 11.9	27.0 ± 5.8	28.6 ± 2.5	17.4 ± 4.0^**^
pmoa	87.9 ± 1.2	73.0 ± 4.3	68.9 ± 5.0	84.1 ± 0.7	52.1 ± 3.1^**^
**Iron reduction and uptake**					
cytochrome	486.0 ± 1.8	397.0 ± 74.5	370.4 ± 74.2	422.1 ± 13.5^**^	271.2 ± 27.8^**^
Cytochrome_cs	288.9 ± 1.0	230.1 ± 40.1	215.5 ± 44.9	254.6 ± 3.4^**^	166.1 ± 13.2^**^
chuT	6.9 ± 0.1	5.2 ± 0.2	5.3 ± 0.1^**^	5.1 ± 0.1^*^	3.9 ± 0.1^**^
ira	0.7 ± 0.0	0.2 ± 0.1	0.0 ± 0.0^**^	0.6 ± 0.0	0.0 ± 0.0^**^
iuc	57.6 ± 0.2	46.1 ± 1.5	46.3 ± 3.0	41.4 ± 0.8^**^	25.7 ± 2.4^*^
mbtD	0.8 ± 0.0	0.2 ± 0.1	0.4 ± 0.1	0.7 ± 0.0	0.0 ± 0.0^**^
mbtF	160.6 ± 0.8	138.6 ± 12.9	116.8 ± 5.4	149.9 ± 1.2	99.2 ± 4.8^*^
mce3	19.5 ± 0.2	19.6 ± 1.1	15.9 ± 0.2^*^	20.0 ± 0.3	20.0 ± 0.5
pchR	2.7 ± 0.03	2.0 ± 0.4	1.8 ± 0.1^*^	3.2 ± 0.03^*^	1.2 ± 0.2
Ni_Fe hydrogenase	15.1 ± 0.4	12.4 ± 1.3	**11.8** ± 1.0^*^	15.6 ± 0.8	12.4 ± 1.1

Data analyzed by t-test, with each incubated sample compared to the pre-incubation sample at the significance level of either

* P < 0.05 or

** *P < 0.01, values marked bold at P < 0.05. amyA, alpha-amylase; pula, pullulanase; ara, homeobox protein araucan; xyla, xylose isomerase; acetglu, acetylglucosaminidase; vdh, vanillin dehydrogenase; glx, hydroxyacylglutathione hydrolase cytoplasmic; mnp, manganese peroxidase; AceA, isocitrate lyase; AceB, malate synthase; aclb, ATP-citrate lyase, beta subunit; AcnA, aconitate hydratase A; frdA_rTCA, fumarate reductase flavoprotein subunit; mdh, malate dehydrogenase; sucD, succinyl-CoA ligase [ADP-forming] subunit alpha; oorA, 2-oxoglutarate:acceptor oxidoreductase, OorA subunit; pgk, phosphoglycerate kinase; PRI, DNA primase; TIM, triosephosphate isomerase, chloroplastic; tktA, transketolase; cdhC_methane, acetyl-CoA decarbonylase/synthase complex; fmdB/fwdB, molybdenum/tungsten formylmethanofuran dehydrogenase; Ftr, formylmethanofuran–tetrahydromethanopterin formyltransferase; hdrB, CoB–CoM heterodisulfide reductase subunit B; Hmd, 5,10-methenyltetrahydromethanopterin hydrogenase; Mch_methane, methenyltetrahydromethanopterin cyclohydrolase; mcra, methyl-coenzyme M reductase I subunit alpha; Mer_methane, 5,10-methylenetetrahydromethanopterin reductase; MT2, metallothionein-2; mtaB, methanol–corrinoid protein co-methyltransferase; mtbC/mttC, dimethylamine corrinoid protein_trimethylamine corrinoid protein; mtmB, monomethylamine methyltransferase MtmB; mttB, trimethylamine methyltransferase MttB; mtxX, putative methyltransferase mtx subunit X; pmoa, methane monooxygenase; mmox, methane monooxygenase component A alpha chain; cytochrome_cs, cytochrome C types; chuT, putative periplasmic hemin-binding protein; ira, Inhibitory regulator protein; mbtD, MBT domain-containing protein; mbtF, peptide synthetase; mce3, MCE-family protein MCE3a; pchR, regulatory protein PchR*.

Anaerobic microbial metabolism requires less energy than aerobic metabolism and should dominate in anoxic or oxygen-limited environments. Microbes relied on anaerobic SOC degradation during the later stages of incubation when functional diversity decreased. In particular, a high acetate content in the organic soil may have played an important role in determining the functional structure, such as that related to methanogenesis (Hines et al., 2008; Herndon et al., 2015; Yang et al., 2016). This finding corroborated our observation that soil type and DOC or organic substrate availability were the major factors influencing microbial diversity (Table 2). Similarly, previous studies suggested that the availability of labile SOC may be more important than temperature dependence for microbial uptake and catabolic capacity in these soils (Jonasson et al., 2004; Kirschbaum, 2006).

### Genes related to CH_4_ production and Fe(III) reduction

Genes affecting CH_4_ cycling, including both methanogen and methanotroph genes, were examined during the incubation experiment. Gene abundance related to methanogenesis decreased in the organic soil at 8°C, and less so in the mineral soil (Figure 3A, Table 3 and Table S4). For example, genes *hdrB, MT2, ftr* and *fmdB/fwdB* all showed a decreasing trend, with significantly lower abundance (*P* < 0.05) after 122 days of incubation at 8°C in the organic soil. Among these, the protein encoded by *hdrB* is known to catalyze the reversible reduction of CoM-S-S-CoB to the thiol-coenzymes H-S-CoM and H-S-CoB. The *fmdB or fwdB* genes encode functionally equivalent subunits of the *formylmethanofuran dehydrogenase*, which catalyzes the reversible oxidation of *N*-formylmethanofuran to form CO_2_ and methanofuran. The *ftr* gene catalyzes the reversible transfer of a formyl group from formylmethanofuran to tetrahydromethanopterin. This decreasing trend of methanogen genes coincides well with decreasing CH_4_ production observed in the organic soil at 8°C (Yang et al., 2016). In contrast, no significant changes in methanogenic gene abundance were observed in the mineral soil over the 122-day incubation (Figure 3, Table S4), which is consistent with a relatively high production rate of CH_4_ maintained in the mineral soil at 8°C (Yang et al., 2016). These observations demonstrate important roles of methanogenic genes in regulating CH_4_ emission in the Arctic tundra soil.

CH_4_ oxidation genes, *pmoA* and *mmox*, also decreased significantly (*P* < 0.05) after 122 days at 8°C in the organic soil (Table 3), consistent with general decrease in aerobic bacteria over time under anoxic conditions. Studies have shown that CO_2_ reduction could be offset by CH_4_ production (Avery et al., 2003), which is usually correlated with soil organic C content at low soil redox conditions (Le Mer and Roger, 2001). Indeed, we found that both CH_4_ oxidation genes (*pmoA* and *mmox*) and C-cycling genes decreased significantly, as microbial fermentation proceeded and organic substrates (e.g., acetate) for methanogenesis accumulated over time (Table 3) (Herndon et al., 2015; Yang et al., 2016). The result also agrees with previous observations that CH_4_ consumption rates are highest in soils where methanogenesis is active (Le Mer and Roger, 2001).

At −2°C, however, CH_4_-related gene abundance was unchanged in the organic soil, but increased slightly in the mineral soil (Figure 3B, Table 3 and Table S4), although none of these changes was significant at *P* < 0.05. These findings are consistent with the experimental observation that CH_4_ production slowly increased, rather than decreasing at near-freezing temperature (Yang et al., 2016). Additionally, the CH_4_ production rate was slightly higher in the mineral soil than in the organic soil at −2°C (Yang et al., 2016), suggesting a lower temperature sensitivity of these processes in the mineral soil than in the organic soil. The same phenomenon was previously observed, and attributed in part to rate-limiting oxidation of propionate and hydrolysis of polysaccharide in these soils (Tveit et al., 2015).

In addition to CH_4_ cycling genes, microbial functional genes associated with iron redox reactions accounted for up to 20.2% of the total number of detected genes. Fe(III) reduction has been demonstrated to be an important process associated with fermentation and methanogenesis in Arctic soils (Herndon et al., 2015; Yang et al., 2016). The abundance of iron cycling genes in the pre-incubation organic soil was higher compared to the mineral soil (Figure 3). During incubation at 8°C, most of the iron reduction genes in the organic soil decreased continuously and significantly from Day 0 to Day 122 (Figure 3A, Table 3), but decreased to a lesser extent in the −2°C incubation (Figure 3B). Notably, genes associated with Fe(III)-reduction, such as those encoding *c*-type cytochromes, decreased substantially (*P* < 0.01) during incubation at 8°C in the organic soil (Table 3). Cytochromes are iron-containing hemeproteins that play a crucial role in metal reduction and electron transfer reactions (Seeliger et al., 1998). This observation coincided with the decreasing trend of C cycling genes (Table 3, Figure 3A), indicating strong coupling between Fe(III) reduction and SOC degradation. Indeed, our results show that labile reducing sugars and ethanol were continuously degraded to form oxidized organic acids (Yang et al., 2016), whereas Fe(III) or iron oxide minerals served as electron acceptors to facilitate SOC degradation. As a result, the ferrous Fe(II) concentration increased continuously during the first 60 days of incubation and remained relatively stable from Day 60 to Day 122 (Table S2) (Yang et al., 2016). In the mineral soil, however, warming did not significantly influence Fe cycling genes (Table S4), possibly due to the availability of abundant Fe(III) or Fe-oxides but low SOC availability in the mineral soil. These findings further indicate important roles played by iron reduction genes and the linkage between SOC availability and iron reduction.

A number of Fe cycling and uptake genes also appeared susceptible to warming since their abundance decreased significantly over time (Table 3). These genes include *chuT*, which encodes a putative periplasmic hemin-binding protein from *Burkholderia pseudomallei K96243* (Holden et al., 2004), and *iuc*, which encodes aerobactin siderophore biosynthesis proteins from *Yersinia mollaretii ATCC 43969* and *Vibrio parahaemolyticus 16* (Tanabe et al., 2003). Additionally, *mbtF* associated with *Mycobacterium smegmatis str. MC2155* (Mohan et al., 2015) decreased substantially (*P* <0.05) after incubation at 8°C. The gene *mbtF* is associated with iron uptake through the biosynthesis of iron-chelating agents (Quadri et al., 1998). It is relatively abundant in the organic soil (Table 3), but virtually absent in the mineral soil (Table S4).

## Conclusions

This work investigated warming effects on microbial functional gene diversity and structure changes during SOC degradation in microcosm incubations with an Arctic tundra soil. We found that microbial communities in the organic soil were distinctly different from those in the mineral soil, irrespective of warming treatments at −2 or 8°C. Production and consumption of labile SOC (e.g., reducing sugar and ethanol) during soil warming incubation proceeded with decrease in microbial functional diversity. Among the ten environmental variables examined, soil type (organic vs. mineral) and organic C substrates showed the strongest correlations with functional gene structure, demonstrating the important influence of labile SOC on the microbial community. Compared with the initial soil, genes associated with C cycling, methanogenesis, and iron metabolism all decreased significantly in the organic soil, but not in the mineral soil at 8°C, and corresponded well with decreases in labile SOC, CH_4_ production, and Fe(III) reduction over time. These results support the coupled pathways between SOC transformation and methanogenesis and Fe(III) reduction (Herndon et al., 2015; Yang et al., 2016) and shed additional light on why SOC in the organic layer of Arctic tundra is vulnerable to climate warming.

## Author contributions

ZY and WF performed the incubation experiment. SY, JV, JZ, and QQ collected and analyzed GeoChip data. ZY, SY, YY, and BG wrote the paper. All authors critically commented on and contributed to the manuscript writing.

### Conflict of interest statement

The authors declare that the research was conducted in the absence of any commercial or financial relationships that could be construed as a potential conflict of interest. The reviewer YD declared a shared affiliation, with no collaboration, with one of the authors YL to the handling Editor.
