# Supplementary material for: Microbial Community and Functional Gene Changes in Arctic Tundra Soils in a Microcosm Warming Experiment
Source: Front Microbiol. 2017 Sep 19;8:1741. doi: 10.3389/fmicb.2017.01741 (PMC5610689; doi:10.3389/fmicb.2017.01741)
Supplement: Supplementary file 1 [file DataSheet1.DOCX]

**SUPPLEMENTARY MATERIAL**

FIGURE S1. Canonical correspondence analysis (CCA) of the microbial community functional composition. The values of CCA1 and CCA2 in the X- and Y-axes are the proportions of total variations of CCA1 and CCA2. Open circles, triangles, and diamonds represent samples incubated at –2 °C for 0, 34 and 122 days, respectively. Solid triangles and diamonds represent samples incubated at 8 °C for 60 and 122 days, respectively. Organic soil samples are marked red, and mineral soils blue. ‘Temp’ represents temperature, ‘DOC’ represents dissolved organic C, ‘Time’ is the incubation time, and ‘Fe’ represents Fe(II)/Fe(total).


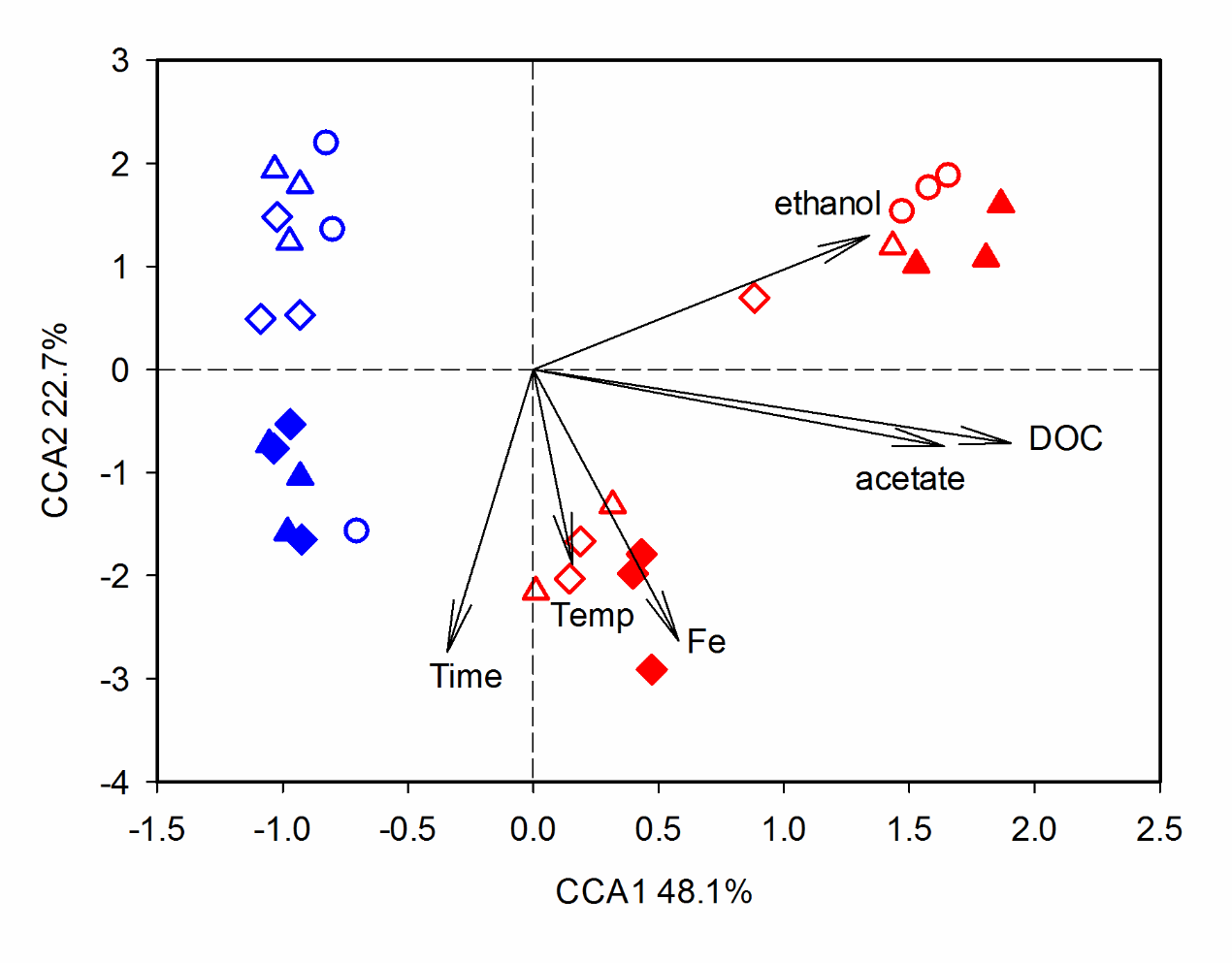


**Table S1**. Dissimilarity tests of GeoChip data sets by MRPP, anosim, and adonis. Distances were calculated by Bray-Curtis.

| \| **Groups** \| \| --- \| \| | **MRPP** | | **anosim** | | **adonis** | |
| --- | --- | --- | --- | --- | --- | --- | --- |
|  | **Delta** | ***P*** | *R* | ***P*** | ***F*** | ***P*** |
| **Whole** | 0.126 | 0.001 | 0.538 | 0.001 | 5.752 | 0.001 |
| **Org vs Min** | 0.161 | 0.002 | 0.220 | 0.001 | 0.115 | 0.027 |

**Table S2**. Production of CH_4_ and CO_2_, and concentrations (μmol g^-1^ dwt) of reducing sugars, ethanol, acetate, and ratios of Fe(II)/Fe(total) during incubation of the organic and mineral layer soils at either –2 °C or 8 °C (Yang et al. 2016a).

|  | | Pre-incubation soil | | Incubated at –2 °C | | | | Incubated at 8 °C | | |
| --- | --- | --- | --- | --- | --- | --- | --- | --- | --- | --- |
|  |  |  |  | day 34 | | day 122 | | day 60 | | day 122 |
| **Organic soil** | | | | | | | | | | |
| Reducing sugar | 14.6±0.8 | | 9.7±0.7 | | 6.2±0.7 | | 6.6±0.8 | | 4.8±0.4 | |
| Ethanol | 7.4±0.4 | | 7.0±0.3 | | 2.2±0.5 | | 2.9±0.5 | | 1.4±0.3 | |
| Acetate | 18.1±1.8 | | 17.4±2.1 | | 5.3±1.2 | | 29.1±2.5 | | 35.7±3.2 | |
| CH_4_ | 0 | | 0.004±0.001 | | 0.045±0.009 | | 7.1±0.5 | | 12.2±1.0 | |
| CO_2_ | 0 | | 5.8±0.7 | | 28.7±3.4 | | 67.2±6.4 | | 87.4±8.3 | |
| Fe(II)/Fe(total) | 0.39±0.02 | | 0.50±0.02 | | 0.61±0.03 | | 0.59±0.04 | | 0.63±0.03 | |
|  |  | |  | |  | |  | |  | |
| **Mineral soil** |  | |  | |  | |  | |  | |
| Reducing sugar | 0.97±0.06 | | 0.58±0.07 | | 0.35±0.06 | | 0.38±0.07 | | 0.32±0.04 | |
| Ethanol | 2.5±0.1 | | 1.8±0.2 | | 0.8±0.2 | | 0.9±0.2 | | 0.6±0.1 | |
| Acetate | 0.58±0.08 | | 0.72±0.10 | | 1.08±0.11 | | 0.13±0.01 | | 0.13±0.01 | |
| CH_4_ | 0 | | 0.08±0.03 | | 0.89±0.22 | | 5.8±0.2 | | 10.8±0.7 | |
| CO_2_ | 0 | | 1.24±0.22 | | 3.93±0.71 | | 12.2±0.80 | | 19.9±1.4 | |
| Fe(II)/Fe(total) | 0.39±0.02 | | 0.46±0.03 | | 0.43±0.02 | | 0.53±0.03 | | 0.47±0.04 | |

**Table S3.** Relationship between iron cycling genes and ten environmental variables (soil type, incubation time, temperature, DOC, reducing sugar, ethanol, acetate, CH_4_, CO_2_, Fe(II)/Fe(total)).

| **Simple Mantel** | ***r*** | ***P**** |
| --- | --- | --- |
| Soil type | 0.159 | **0.035** |
| Time | 0.092 | 0.083 |
| Temperature | 0.070 | 0.083 |
| DOC | 0.170 | **0.035** |
| Reducing Sugar | 0.090 | 0.183 |
| Ethanol | 0.048 | 0.270 |
| Acetate | 0.280 | **0.040** |
| CH_4_ | 0.178 | 0.083 |
| CO_2_ | 0.252 | 0.083 |
| Fe(II)/Fe(total) | 0.125 | 0.116 |

* *P* values were adjusted by the Benjamini-Hochberg method, using sequentially modified Bonferroni correction for multiple hypothesis testing to avoid the false Discovery Rate (FDR).

**Table S4**. Total relative gene abundances related to carbon degradation, fermentation, CH_4_ cycling, and iron reduction and uptake in the mineral soil after incubation at either –2°C or 8°C. Data analyzed by *t*-test, with each incubated sample compared to the pre-incubation sample at the significance level of either **P* < 0.05 or ***P* < 0.01.

| Genes (in thousands) | | Pre-incubation soil | | Incubated at –2 °C | | | | Incubated at 8 °C | | |
| --- | --- | --- | --- | --- | --- | --- | --- | --- | --- | --- |
|  |  |  |  | day 34 | | day 122 | | day 60 | | day 122 |
| **Carbon degradation** (Labile to recalcitrant from Starch to Lignin) | | | | | | | | | | |
| **Starch** | | | | | | | | | | |
| amyA | 6404.7±568.8 | | 7359.2±386.8 | | 7197.6±164.7 | | 5803.9±164.7 | | 6018.3±109.2 | |
| glucoamylase | 286.6±24.1 | | 341.0±23.5 | | 345.1±15.7 | | 268.2±15.7 | | 284.9±11.8 | |
| pula | 265.8±24.4 | | 311.1±21.1 | | 306.7±8.5 | | 228.7±8.5 | | 242.2±6.3 | |
| **Hemicellulose** |  | |  | |  | |  | |  | |
| ara | 977.3±370 | | 1086.5±48.6 | | 1061.0±22.2 | | 902.8±22.2 | | 937.5±17.5 | |
| xyla | 484.6±48.0 | | 562.6±32.4 | | 547.0±13.8 | | 412.1±13.8 | | 430.4±12.2 | |
| xylanase | 723.6±75.9 | | 875.1±43.5 | | 821.3±48.8 | | 639.8±48.8 | | 686.7±14.9 | |
| **Cellulose** |  | |  | |  | |  | |  | |
| cellobiase | 523.1±70.9 | | 618.9±30.0 | | 600.8±17.6 | | 465.4±17.6 | | 467.8±18.7 | |
| endoglucanase | 449.8±47.7 | | 533.2±32.4 | | 512.9±16.7 | | 385.6±16.7 | | 414.0±9.1 | |
| exoglucanase | 131.6±17.2 | | 163.2±8.6 | | 149.6±9.2 | | 112.8±9.2 | | 123.5±5.2 | |
| **Chitin** |  | |  | |  | |  | |  | |
| acetyl-glucosaminidase | 1106.8±106.9 | | 1299.2±91.7 | | 1248.3±2.8 | | 963.8±2.8 | | 993.4±29.1 | |
| chitinase | 1912.3±190.5 | | 2161.6±128.5 | | 2132.2±36.4 | | 1662.9±36.4 | | 1714.4±44.3 | |
| **Aromatic** |  | |  | |  | |  | |  | |
| limeh | 204.6±24.5 | | 242.8±11.8 | | 230.8±8.6 | | 183.1±8.6 | | 183.8±3.0 | |
| vdh | 85.7±8.9 | | 99.0±6.1 | | 94.7±40 | | 74.5±4.0 | | 80.7±4.5 | |
| **Lignin** |  | |  | |  | |  | |  | |
| glx | 80.6±11.2 | | 95.4±3.9 | | 95.2±0.4 | | 75.5±0.4 | | 74.0±5.6 | |
| mnp | 155.8±18.0 | | 179.4±11.4 | | 181.1±10.5 | | 129.0±10.5 | | 137.0±1.6 | |
| phenol_  oxidase | 367.6±49.9 | | 434.3±21.5 | | 416.1±16.7 | | 325.6±16.7 | | 342.1±17.7 | |
| **Fermentation** | | | | | | | | | | |
| AceA | 433.5±49.3 | | 491.5±31.9 | | 487.7±47.1 | | 373.4±11.0 | | 380.9±17.1 | |
| AceA_fungi | 13.6±1.5 | | 17.5±0.9 | | 16.6±1.8 | | 14.6±1.4 | | 14.0±0.4 | |
| AceB | 928.3±89.4 | | 1086.1±63.3 | | 1055.6±98.8 | | 820.4±15.6 | | 836.3±23.0 | |
| AceB_fungi | 17.6±2.9 | | 24.0±0.9 | | 20.5±1.8 | | 17.3±0.2 | | 19.0±0.3 | |
| aclb | 39.9±7.1 | | 50.4±2.3 | | 47.6±4.0 | | 32.9±1.5 | | 33.2±2.8 | |
| AcnA | 34.9±4.0 | | 41.2±2.0 | | 41.2±3.2 | | 32.9±0.5 | | 31.9±1.3 | |
| frdA_rTCA | 55.3±7.5 | | 65.6±4.3 | | 63.1±6.9 | | 40.6±2.8 | | 45.1±2.5 | |
| mdh | 90.9±12.3 | | 100.4±4.8 | | 102.5±4.9 | | 73.4±4.7 | | 69.3±4.7 | |
| sucD | 12.8±2.0 | | 15.2±0.9 | | 15.5±1.9 | | 11.4±0.7 | | 11.9±1.3 | |
| oorA | 8.4±0.3 | | 9.6±1.7 | | 9.0±1.0 | | **6.3±0.2**** | | **6.5±0.3**** | |
| pgk | 306.5±41.9 | | 365.2±16.3 | | 351.6±28.6 | | 266.4±15.1 | | 286.5±7.8 | |
| PRI | 450.6±54.2 | | 546.1±26.8 | | 519.1±38.2 | | 387.1±7.7 | | 402.2±6.4 | |
| TIM | 686.6±31.5 | | 775.0±43.5 | | 800.6±73.9 | | 652.0±5.2 | | 665.9±14.1 | |
| tktA | 1084.6±105.2 | | 1249.0±49.0 | | 1222.7±106.4 | | 963.1±26.2 | | 1024.4±25.8 | |
| **CH_4_ cycling** | | | | | | | | | | |
| **Methanogenesis** | | | | | | | | | | |
| cdhC methane | 2.6±0.3 | | 3.0±0.2 | | 2.7±0.2 | | **1.4±0.2*** | | 1.8±0.02 | |
| fmdB/fwdB | 86.7±8.4 | | 105.6±7.8 | | 97.6±7.1 | | 62.0±4.8 | | 70.1±0.4 | |
| Ftr | 84.0±11.0 | | 98.7±4.7 | | 96.6±6.6 | | 73.2±5.0 | | 66.6±1.1 | |
| hdrB | 298.4±9.3 | | 342.1±20.6 | | 355.6±34.4 | | **259.1±4.5*** | | 270.0±1 | |
| Hmd | 1.3±0.3 | | 1.8±0.1 | | 1.8±0.1 | | 1.1±0.1 | | 0.9±0.2 | |
| Mch_methane | 39.2±5.0 | | 41.2±1.9 | | 43.8±3.9 | | 34.9±0.8 | | 35.9±3.4 | |
| mcrA | 243.7±18.8 | | 232.9±15.2 | | 239.3±16.2 | | 276.1±4.8 | | 281.5±4.7 | |
| Mer_methane | 23.2±3.5 | | 30.1±2.0 | | 27.5±1.3 | | 19.4±1.4 | | 20.3±0.7 | |
| mrtH | 50.7±6.5 | | 63.7±5.7 | | 61.0±5.4 | | 38.7±1.5 | | 42.2±1.6 | |
| MT2 | 4.3±1.3 | | 5.3±0.6 | | 4.9±0.3 | | 4.5±0.3 | | 4.4±0.3 | |
| mtaB | 6.7±0.7 | | 9.0±0.2 | | 8.4±0.5 | | 6.2±0.5 | | 5.6±0.4 | |
| mtbC/mttC | 3.9±0.7 | | 4.3±0.2 | | 4.0±0.1 | | 3.3±0.2 | | 3.4±0.2 | |
| mtmB | 3.0±0.1 | | 4.2±0.02 | | 3.9±0.03 | | 1.9±0.0 | | 2.0±0.1 | |
| mttB | 0.3±0.3 | | 0.2±0.2 | | 0.3±0.5 | | 0.00±0.04 | | 0.1±0.1 | |
| mtxX | 2.2±0.1 | | 2.7±0.0 | | 2.7±0.1 | | 1.4±0.0 | | 1.5±0.0 | |
| **Methane oxidation** |  | |  | |  | |  | |  | |
| mmox | 32.5±6.5 | | 40.3±5.7 | | 35.3±5.4 | | 36.4±1.5 | | 33.0±1.6 | |
| pmoa | 59.2±5.0 | | 70.8±2.1 | | 67.3±3.4 | | 49.2±2.9 | | 52.9±0.3 | |
| **Iron reduction and uptake** | | | | | | | | | | |
| cytochrome | 362.9±46.6 | | 423.9±20.3 | | 413.4±40.8 | | 314.8±20.1 | | 332.9±16.2 | |
| Cytochrome_cs | 178.8±24.1 | | 212.2±17.5 | | 208.6±18.9 | | 151.9±3.9 | | 150.9±8.0 | |
| chuT | 6.7±0.9 | | 8.8±1.1 | | **9.0±0.5**** | | 5.4±0.8 | | 5.9±0.3 | |
| ira | 6.3±0.2 | | 8.6±0.2 | | 8.4±0.1 | | 6.2±0.02 | | 6.4±0.01 | |
| iuc | 0.3±0.1 | | 0.34±0.06 | | 0.3±0.01 | | 0.4±0.1 | | 0.0±0.0 | |
| mbtD | 59.8±4.4 | | 71.6±2.4 | | 65.2±3.3 | | 63.1±1.8 | | 63.8±1.7 | |
| mbtF | 0.2±0.1 | | 0.0±0.0 | | 0.0±0.0 | | 0.0±0.0 | | 0.0±0.0 | |
| mce3 | 130.2±7.0 | | 153±3.7 | | 141.8±5.8 | | 106.2±3.5 | | 120.6±0.9 | |
| pchR | 8.3±0.6 | | 10.2±0.3 | | 11.2±0.4 | | 8.8±0.4 | | 9.3±0.3 | |
| Ni_Fe hydrogenase | 0.9±0.1 | | 1.5±0.1 | | 1.6±0.1 | | 1.0±0.2 | | 1.0±0.2 | |

Note: amyA = alpha-amylase; pula = pullulanase; ara = homeobox protein araucan; xyla = xylose isomerase; acetglu = acetylglucosaminidase; vdh = vanillin dehydrogenase; glx = hydroxyacylglutathione hydrolase cytoplasmic; mnp = manganese peroxidase; AceA = isocitrate lyase; AceB = malate synthase; aclb = ATP-citrate lyase, beta subunit; AcnA = aconitate hydratase A; frdA_rTCA = fumarate reductase flavoprotein subunit; mdh = malate dehydrogenase; sucD = succinyl-CoA ligase [ADP-forming] subunit alpha; oorA = 2-oxoglutarate:acceptor oxidoreductase, OorA subunit; pgk = phosphoglycerate kinase; PRI = DNA primase; TIM = triosephosphate isomerase, chloroplastic; tktA = transketolase; cdhC_methane = acetyl-CoA decarbonylase/synthase complex; fmdB/fwdB = molybdenum/tungsten formylmethanofuran dehydrogenase; Ftr = formylmethanofuran-tetrahydromethanopterin formyltransferase; hdrB = CoB--CoM heterodisulfide reductase subunit B; Hmd = 5,10-methenyltetrahydromethanopterin hydrogenase; Mch_methane = methenyltetrahydromethanopterin cyclohydrolase; mcra = methyl-coenzyme M reductase I subunit alpha; Mer_methane = 5,10-methylenetetrahydromethanopterin reductase; MT2 = metallothionein-2; mtaB = methanol--corrinoid protein co-methyltransferase; mtbC/mttC = dimethylamine corrinoid protein/trimethylamine corrinoid protein; mtmB = monomethylamine methyltransferase MtmB; mttB = trimethylamine methyltransferase MttB; mtxX = putative methyltransferase mtx subunit X; pmoa = methane monooxygenase; mmox = methane monooxygenase component A alpha chain; cytochrome_cs = cytochrome C types; chuT = putative periplasmic hemin-binding protein; ira = Inhibitory regulator protein; mbtD = MBT domain-containing protein; mbtF = peptide synthetase; mce3 = MCE-family protein MCE3a; pchR = regulatory protein PchR.
